# The Prognostic Value of Troponin-T in Out-of-Hospital Cardiac Arrest Without ST-Segment Elevation: A COACT Substudy

**DOI:** 10.1016/j.jscai.2023.101191

**Published:** 2023-10-14

**Authors:** Eva M. Spoormans, Jorrit S. Lemkes, Gladys N. Janssens, Nina W. van der Hoeven, Lucia S.D. Jewbali, Eric A. Dubois, Martijn Meuwissen, Tom A. Rijpstra, Hans A. Bosker, Michiel J. Blans, Gabe B. Bleeker, Remon Baak, Georgios J. Vlachojannis, Bob J.W. Eikemans, Pim van der Harst, Iwan C.C. van der Horst, Michiel Voskuil, Joris J. van der Heijden, Albertus Beishuizen, Martin Stoel, Cyril Camaro, Hans van der Hoeven, José P. Henriques, Alexander P.J. Vlaar, Maarten A. Vink, Bas van den Bogaard, Ton A.C.M. Heestermans, Wouter de Ruijter, Thijs S.R. Delnoij, Harry J.G.M. Crijns, Pranobe V. Oemrawsingh, Marcel T.M. Gosselink, Koos Plomp, Michael Magro, Paul W.G. Elbers, Stéphanie van der Pas, Niels van Royen

**Affiliations:** a Department of Cardiology, Amsterdam University Medical Center, location VUmc, Amsterdam, the Netherlands; b Department of Cardiology, Erasmus Medical Center, Rotterdam, the Netherlands; c Department of Intensive Care Medicine, Erasmus Medical Center, Rotterdam, the Netherlands; d Department of Cardiology, Amphia Hospital, Breda, the Netherlands; e Department of Intensive Care Medicine, Amphia Hospital, Breda, the Netherlands; f Department of Cardiology, Rijnstate Hospital, Arnhem, the Netherlands; g Department of Intensive Care Medicine, Rijnstate Hospital, Arnhem, the Netherlands; h Department of Cardiology, HAGA Hospital, Den Haag, the Netherlands; i Department of Intensive Care Medicine, HAGA Hospital, Den Haag, the Netherlands; j Department of Cardiology, Maasstad Hospital, Rotterdam, the Netherlands; k Department of Cardiology, University Medical Center Utrecht, Utrecht, the Netherlands; l Department of Intensive Care Medicine, Maasstad Hospital, Rotterdam, the Netherlands; m Department of Cardiology, University Medical Center Groningen, Groningen, the Netherlands; n Department of Intensive Care Medicine, University Medical Center Groningen, Groningen, the Netherlands; o Department of Intensive Care Medicine, Maastricht University Medical Center, Maastricht, the Netherlands; p Department of Intensive Care Medicine, University Medical Center Utrecht, Utrecht, the Netherlands; q Intensive Care Center, Medisch Spectrum Twente, Enschede, The Netherlands; r Department of Cardiology, Medisch Spectrum Twente, Enschede, The Netherlands; s Department of Cardiology, Radboud University Medical Center, Nijmegen, the Netherlands; t Department of Intensive Care Medicine, Radboud University Medical Center, Nijmegen, the Netherlands; u Department of Cardiology, Amsterdam University Medical Center, location AMC, Amsterdam, the Netherlands; v Department of Intensive Care Medicine, Amsterdam University Medical Center, location AMC, Amsterdam, the Netherlands; w Department of Cardiology, OLVG, Amsterdam, the Netherlands; x Department of Intensive Care Medicine, OLVG, Amsterdam, the Netherlands; y Department of Cardiology, Noord West Ziekenhuisgroep, Alkmaar, the Netherlands; z Department of Intensive Care Medicine, Noord West Ziekenhuisgroep, Alkmaar, the Netherlands; aa Department of Cardiology, Maastricht University Medical Center, Maastricht, the Netherlands; bb Department of Cardiology, Haaglanden Medical Center, Den Haag, the Netherlands; cc Department of Cardiology, Isala Hospital, Zwolle, the Netherlands; dd Department of Cardiology, Tergooi Hospital, Blaricum, the Netherlands; ee Department of Cardiology, Elisabeth-Tweesteden Hospital, Tilburg, the Netherlands; ff Department of Intensive Care Medicine, Amsterdam University Medical Center, location VUmc, Amsterdam, the Netherlands; gg Epidemiology and Data Science, Amsterdam University Medical Center, location Vrije Universiteit Amsterdam, Amsterdam, Netherlands; hh Amsterdam Public Health, Methodology, Amsterdam, The Netherlands

**Keywords:** acute coronary syndrome, coronary angiography, out-of-hospital cardiac arrest

## Abstract

**Background:**

In out-of-hospital cardiac arrest (OHCA) without ST-elevation, predictive markers that can identify those with a high risk of acute coronary syndrome are lacking.

**Methods:**

In this post hoc analysis of the Coronary Angiography after Cardiac Arrest (COACT) trial, the baseline, median, peak, and time-concentration curves of troponin-T (cTnT) (T-AUC) in OHCA patients without ST-elevation were studied. cTnT values were obtained at predefined time points at 0, 3, 6, 12, 24, 36, 28, and 72 hours after admission. All patients who died within the measurement period were not included. The primary outcome was the association between cTnT and 90-day survival. Secondary outcomes included the association of cTnT and acute thrombotic occlusions, acute unstable lesions, and left ventricular function.

**Results:**

In total, 352 patients were included in the analysis. The mean age was 64 ± 13 years (80.4% men). All cTnT measures were independent prognostic factors for mortality after adjustment for potential confounders age, sex, history of coronary artery disease, witnessed arrest, time to BLS, and time to return of spontaneous circulation (eg, for T-AUC: hazard ratio, 1.44; 95% CI, 1.06-1.94; *P* = .02; *P* value for all variables ≤.02). Median cTnT (odds ratio [OR], 1.58; 95% CI, 1.18-2.12; *P* = .002) and T-AUC (OR, 2.03; 95% CI, 1.25-3.29; *P* = .004) were independent predictors for acute unstable lesions. Median cTnT (OR, 1.62; 95% CI, 1.17-2.23; *P* = .003) and T-AUC (OR, 2.16; 95% CI, 1.27-3.68; *P* = .004) were independent predictors for acute thrombotic occlusions. CTnT values were not associated with the left ventricular function (eg, for T-AUC: OR, 2.01; 95% CI, 0.65-6.19; *P* = .22; *P* value for all variables ≥.14)

**Conclusion:**

In OHCA patients without ST-segment elevation, cTnT release during the first 72 hours after return of spontaneous circulation was associated with clinical outcomes.

## Highlights

• Troponin-T release in the first days after return of spontaneous circulation was associated with survival.
• Patients with significant coronary artery disease had higher troponin T values.
• The performance of troponin T values in predicting survival was poor.
• The performance in predicting acute coronary syndrome, troponin T had a fair discrimination ability.

## Central Illustration

**Figure:**
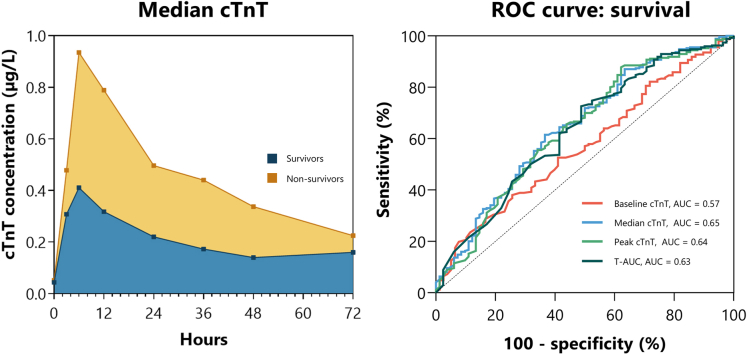

## Introduction

In Europe, the annual incidence of out-of-hospital cardiac arrest (OHCA) is between 67 to 170 patients per 10,000 inhabitants.^1^ Despite major advances in postarrest care, survival in these patients remains poor.^2^ Early identification and treatment of reversible causes of the arrest are paramount to improve survival rates. Ischemic heart disease is thought to be the most common cause of the arrest as ∼70% of patients have clinically significant coronary artery disease (CAD).^3^ Among patients without ST-segment elevation after the return of spontaneous circulation (ROSC), unselected immediate coronary angiography is not superior over a delayed invasive strategy, as recently demonstrated in 4 randomized trials.^4^, ^5^, ^6^, ^7^, ^8^ Immediate coronary angiography, however, may benefit those with acute coronary syndrome (ACS), which was equivalent to a rate of 15% of patients having an acute unstable lesion as seen in the Coronary Angiography after Cardiac Arrest (COACT) trial.^4^ Identifying those without ST-segment elevation after OHCA in whom immediate revascularization may alleviate myocardial infarction is challenging. Prior studies have demonstrated that ischemic electrocardiogram (ECG) patterns, such as ST-segment depression or T-wave inversion, do not facilitate the identification of this patient group.^9^^,^^10^ Furthermore, prodromal symptoms indicating ACS are often hard to discern since most of the OHCA patients are comatose. Therefore, a predictive marker that can identify those with a high risk of ACS is needed among patients without ST-segment elevation after resuscitation. Troponin T (cTnT), a highly sensitive indicator of myocardial injury, is routinely obtained in clinical practice to discriminate between ACS and non-ACS.^11^ However, cTnT concentrations obtained directly after ROSC have shown to be a poor predictor for acute coronary lesions, limiting their contribution to clinical decision-making.^12^, ^13^, ^14^ Whether the course of cTnT concentrations, obtained by serial cTnT measurements, is associated with ACS due to an acute coronary lesion and concomitant survival in OHCA patients without ST-segment elevation has not yet been established. Therefore, we performed a post hoc analysis using the database from a multicenter, randomized trial of OHCA patients without ST-segment elevation undergoing immediate or delayed coronary angiography^4^ to evaluate multiple cTnT values over time and its association with survival, neurologic outcome, and acute CAD.

## Methods

### Study design

This is a post hoc analysis of the COACT trial.^4^ In brief, the COACT study was a randomized controlled, multicenter trial that investigated the benefit of immediate coronary angiography compared to delayed angiography after neurological recovery in OHCA patients. Comatose patients successfully resuscitated from cardiac arrest without ST-segment elevation were eligible for the trial. Key exclusion criteria were: signs of ST-segment elevation myocardial infarction (STEMI) on the postresuscitation ECG, an obvious or suspected noncoronary cause of the arrest, recurrence of ventricular tachycardia, and shock. A full list of in and exclusion criteria has been reported previously.^4^ Postresuscitation care was according to the resuscitation guidelines.^2^ For this post hoc analysis, we aimed to assess the value of cTnT in the first days after the arrest in predicting clinical outcomes. cTnT values were drawn at prespecified time points from admission to 72 hours after the arrest. The first cTnT value obtained at the emergency department after ROSC (ie, baseline cTnT), the median cTnT, the peak cTnT, and the time-concentration curve (T-AUC) were evaluated for each patient. We included only patients who had survived until 72 hours in this study. The primary outcome of this study was the association between cTnT and 90-day survival. Secondary outcomes included the association of cTnT and neurologic outcome, acute unstable lesions, acute thrombotic occlusions, and left ventricular function (LVF). The trial design of the main COACT trial was reviewed and approved by the VUMC ethics committee and is registered at the Netherlands Trial Register, number NTR4973.

### Neurological outcomes

Neurological outcomes were defined by Cerebral Performance Category (CPC) scores. CPC scores were determined at 90 days after the arrest. A favorable neurological outcome was defined by a CPC score of 1 or 2; a CPC score of 3 to 5 indicated a poor neurological outcome. Definitions of CPC scores are described in the Supplemental Appendix.

### Signs of ischemia on ECG

Signs of ischemia on ECG are defined as depressions of 1 mm or more in 2 contiguous leads, or T-wave inversion in 2 contiguous leads, or both.

### Acute unstable lesions and acute thrombotic occlusions

Acute unstable lesions were defined as all coronary lesions with a stenosis severity of ≥70% and the presence of characteristics of plaque disruption including lesion irregularity, dissection, haziness, or thrombus defined by coronary angiography. Acute thrombotic occlusions were defined as coronary lesions with a recent thrombus leading to total occlusion of the vessel.

### cTnT measurements

Venous blood samples for cTnT (μg/L) were obtained at 0, 3, 6, 12, 24, 36, 48, and 72 hours after admission. Median cTnt was the median cTnT value per patient during the first 72 hours. Peak cTnT was the highest cTnT measurement for each patient. The baseline cTnT was the cTnT measurement obtained directly on the arrival at the emergency department after ROSC. In addition to baseline cTnT, median cTnT value, and peak cTnT value, the time-concentration curve for each patient was calculated as a representation of the extent of myocardial damage.

### LVF

LVF and left ventricular ejection fraction (LVEF) were assessed in all patients who underwent cardiac magnetic resonance or echocardiography during hospitalization.

### Statistical analysis

Continuous variables are expressed as mean ± SD or median (IQR). Categorical variables were expressed in numbers. T-AUC was calculated by connecting all available consecutive measurements by straight line segments and computing the area of the resulting polygon. To assess the association between baseline cTnT, median cTnT, peak cTnT (per unit increase of each cTnT measurement) and T-AUC and survival from 72 hours, hazard ratios (HR) and their 95% CI were derived using Cox regression. Cox regression was then used to adjust the HR for baseline cTnT, median cTnT, peak cTnT, and T-AUC on survival for the following potential confounders: age, sex, history of coronary artery disease, witnessed arrest, time to basic life support, and time to ROSC. Odds ratios (OR) and their 95% CI were used to assess the association between baseline cTnT, median cTnT, peak cTnT, and T-AUC and favorable/poor neurologic outcomes, acute unstable lesions, acute thrombotic occlusions and LVF using logistic regression. To determine the clinical performance of the previously mentioned cTnT values, a receiver operating characteristic (ROC) curve and their area under the curve (AUC) was determined. Spearman's rank correlation was computed to assess the relationship between LVEF and median cTnT, peak cTnT, and T-AUC. Mann-Whitney *U* test or Kruskal-Wallis test was used to compare the area under the time-concentration curve values between groups (survivors/nonsurvivors, significant CAD/nonsignificant CAD/unstable lesion as identified on coronary angiography). A *P* value of <0.05 was considered statistically significant. Statistical analysis was performed using SPSS Statistics, Version 28.0 (IBM Corp).

## Results

### Patients

**Figure 1:**
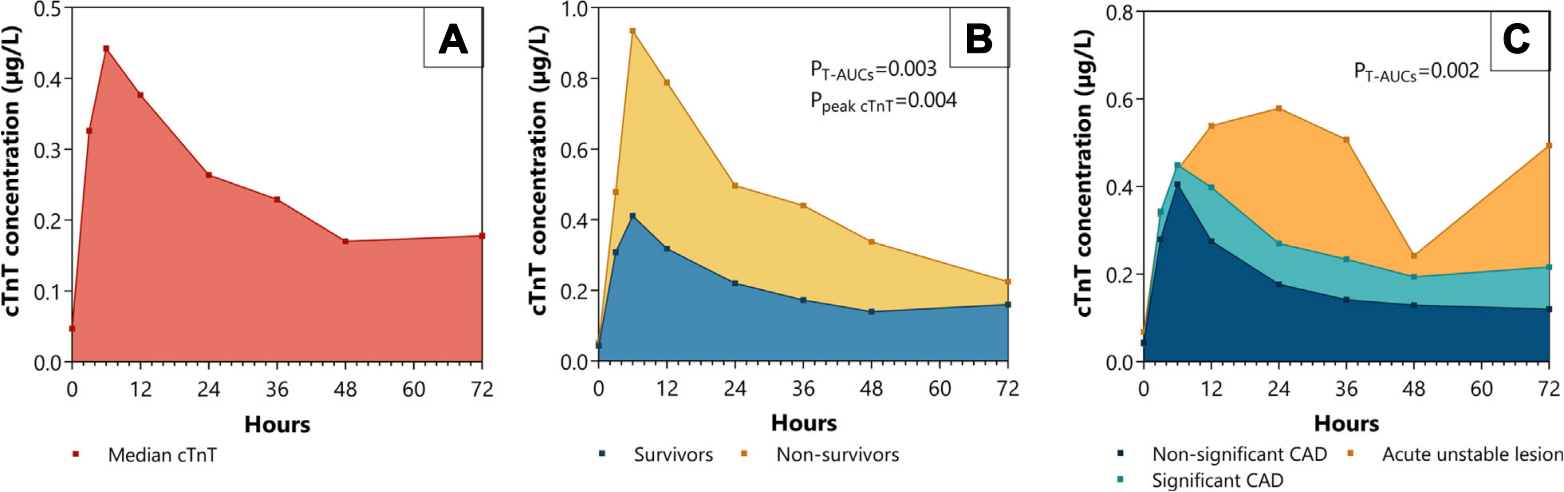
**Troponin T (cTnT) concentrations.** (**A**) Median cTnT values in the first 72 hours of admission. (**B**) Median cTnT values in cardiac arrest survivors and nonsurvivors. (**C**) Median cTnT values in patients with nonsignificant coronary artery disease (CAD), significant CAD, and acute unstable lesions.

Between January 2015 and July 2018, 552 OHCA patients with shockable rhythm and absence of ST-segment elevation were included in the COACT trial. In total, 417 patients had serial cTnT measurements, of whom 57 patients died within the measurement period of 72 hours, leaving 360 patients eligible for analysis (Supplemental Figure S1). Eight patients had extremely large T-AUC values (range T-AUC 15.679-173.074) compared to the range of the T-AUC of the remaining 352 patients (T-AUC 0.002-4.491). In all of these 8 patients, this was caused by 1 or 2 cTnT measurements that disproportionally diverged from the rest of the measurements within the curve. Therefore, these patients were considered outliers and removed from primary analysis out of concern for violation of the linearity and proportional hazard assumptions underlying the Cox regression. A histogram showing the distribution of T-AUC is shown in Supplemental Figure S2, and outcome measures including these 8 outliers are depicted in Supplemental Table S1. Of the 352 patients in the analysis, 283 (80.4%) were men, the mean age was 64 ± 13 years, and the median cTnT at baseline was 0.047 μg/L (0.027-0.095 μg/L) (Table 1). Signs of ischemia on ECG were present in 223/336 (66.4%) of patients, and the median Glasgow Coma Score at admission was 3 (3-3). Peak levels of cTnT were reached between 3 to 12 hours, and then steadily decreased (Figure 1A). Both peak levels of cTnT (P_peak cTnT_ = 0.004) and median cTnT values were higher in patients who did not survive until 90 days (*P* = .003) (Figure 1B and Central Illustration). Patients with acute unstable lesions, as identified on coronary angiography, had higher peak levels of cTnT (*P* = .04) and median cTnT values compared to patients with stable significant CAD or nonsignificant CAD (*P* = .002) (Figure 1C).

**Table 1:** Baseline characteristics.

	Patients (N = 352)
Male sex	283 (80.4)
Age, y	64 ± 13
Medical history	
Diabetes mellitus	56/351 (16.0)
Hypertension	161/350 (46.0)
Hypercholesterolemia	89/350 (25.4)
Peripheral artery disease	25/351 (7.1)
Cerebrovascular accident	20/351 (5.7)
Smoker	86/327 (26.3)
Myocardial infarction	87 (24.7)
Coronary artery disease	120 (34.1)
Percutaneous coronary intervention	65/351 (18.5)
Coronary artery bypass grafting	39/351 (11.1)
Arrest characteristics	
Arrest witnessed	292 (83.0)
Time arrest to BLS, min	2 (1-5)
Time arrest to ROSC, min	12 (7-20)
Signs of ischemia on ECG^a^	223/336 (66.4)
GCS at admission^b^	3 (3-3)
Laboratory values	
Troponin T, μg/L	0.047 (0.027-0.095)
CK-MB, μg/L	5.9 (3.7-17.5)
Lactate, mmol/L	5.4 ± 3.4

Values are mean ± SD, n/N (%), or median (IQR). BLS, basic life support; CK-MB, creatine kinase-myoglobin binding; ECG, electrocardiogram; GCS Glasgow coma scale; OHCA, out-of-hospital cardiac arrest; ROSC, return of spontaneous circulation.

a Ischemia on the ECG was defined as ST-depression of ≥0.1 mV, T-wave inversion in ≥2 contiguous leads, or both.

b Glasgow Coma Scale is the summation of eye, verbal, and motor responses and ranges from 3 (deep coma) to 15 (responsive).

**Central Illustration:**
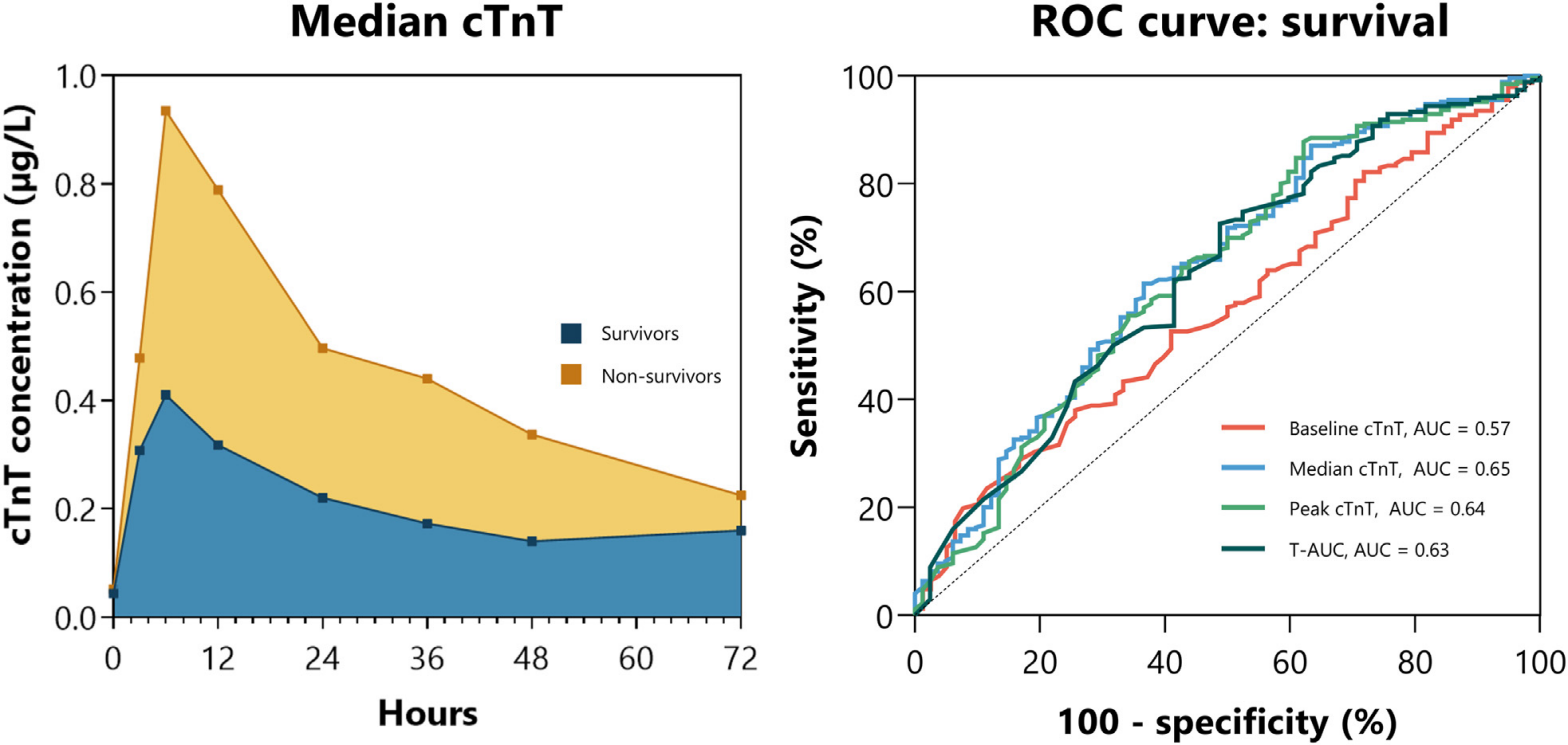
**Troponin T (cTnT) concentrations and Area under the receiver operating characteristic (ROC) curve.** Median cTnT values in cardiac arrest survivors and nonsurvivors. ROC for serial troponin T (cTnT) values and survival.

### Survival and neurologic outcome

**Figure 2:**
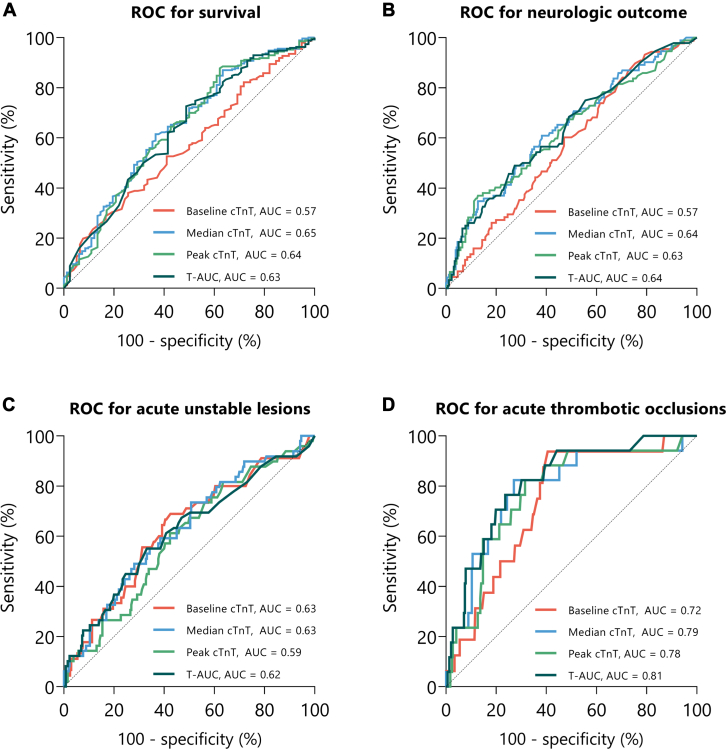
**Area under the receiver operating characteristic (ROC) curve.** (**A**) ROC for serial troponin T (cTnT) values and survival. (**B**) ROC for serial cTnT values and neurologic outcome. (**C**) ROC curve for serial cTnT values and acute unstable lesions. (**D**) ROC curve for serial cTnT values and acute thrombotic occlusions.

At 90 days, 270/352 (76.7%) patients were alive. As shown in Table 2, baseline cTnT (unadjusted HR for death, 1.64; 95% CI, 1.16-2.31; *P* = .005; adjusted HR for death, 2.05; 95% CI, 1.47-2.86; *P* < .001), median cTnT (unadjusted HR for death, 1.28; 95% CI, 1.12-1.46; *P* < .001; adjusted HR for death, 1.30; 95% CI, 1.11-1.53; *P* = .001), peak cTnT (unadjusted HR for death, 1.10; 95% CI, 1.03-1.17; *P* = .005; adjusted HR for death, 1.09; 95% CI, 1.01-1.18; *P* = .02) and T-AUC (unadjusted HR for death, 1.35; 95% CI, 1.07-1.72; *P* = .01; adjusted HR for death, 1.44; 95% CI, 1.06-1.94; *P* = .02) were all independent prognostic factors for mortality. The median cTnT (unadjusted OR, 1.42; 95% CI, 1.14-1.77; *P* = .001; adjusted OR, 1.47; 95% CI, 1.12-1.94; *P* = .006), peak cTnT (unadjusted OR, 1.17; 95% CI, 1.04-1.31; *P* = .007; adjusted OR, 1.18; 95% CI, 1.02-1.36; *P* = .03) and T-AUC (unadjusted OR, 1.66; 95% CI, 1.16-2.38; *P* = .006; adjusted OR, 1.84; 95% CI, 1.16-2.91; *P* = .01) were all independently associated with a poor neurological outcome. This was not the case for baseline cTnT (unadjusted OR, 1.44; 95% CI, 0.81-2.58; *P* = .22, adjusted OR, 2.67; 95% CI, 0.90-7.87; *P* = .08). As shown in Figure 2A and B and the Central Illustration, all cTnT values performed poorly in predicting survival and neurological outcomes (AUC between 0.57-0.65).

**Table 2:** The association between troponin T (cTnT) and primary and secondary outcomes.

		Baseline cTnT		Median cTnT		Peak cTnT		T-AUC	
		Effect size (95% CI)	*P* value	Effect size (95% CI)	*P* value	Effect size (95% CI)	*P* value	Effect size (95% CI)	*P* value
Survival^a^	Unadjusted	1.64 (1.16-2.31)	.005	1.28 (1.12-1.46)	<.001	1.10 (1.03-1.17)	.005	1.35 (1.07-1.72)	.01
	Adjusted	2.05 (1.47-2.86)	<.001	1.30 (1.11-1.53)	.001	1.09 (1.01-1.18)	.02	1.44 (1.06-1.94)	.02
Poor neurological outcome^b^	Unadjusted	1.44 (0.81-2.58)	.22	1.42 (1.14-1.77)	.001	1.17 (1.04-1.31)	.007	1.66 (1.16-2.38)	.006
	Adjusted	2.67 (0.90-7.87)	.08	1.47 (1.12-1.94)	.006	1.18 (1.02-1.36)	.03	1.84 (1.16-2.91)	.01
Acute unstable lesions^b^	Unadjusted	2.13 (0.92-4.91)	.08	1.39 (1.11-1.73)	.004	1.11 (0.99-1.24)	.09	1.74 (1.20-2.53)	.004
	Adjusted	3.32 (1.11-9.94)	.03	1.58 (1.18-2.12)	.002	1.14 (0.99-1.30)	.06	2.03 (1.25-3.29)	.004
Acute thrombotic occlusions^b^	Unadjusted	2.09 (1.00-4.34)	.05	1.55 (1.20-2.00)	<.001	1.17 (1.02-1.34)	.02	2.18 (1.43-3.34)	<.001
	Adjusted	2.38 (0.96-5.91)	.06	1.62 (1.17-2.23)	.003	1.16 (0.99-1.36)	.06	2.16 (1.27-3.68)	.004
Abnormal left ventricular function^b^	Unadjusted	1.54 (0.43-5.53)	.51	2.06 (0.79-5.36)	.14	1.11 (0.88-1.39)	.38	2.01 (0.65-6.19)	.22
	Adjusted	1.89 (0.41-8.66)	.41	1.89 (0.48-7.44)	.36	0.98 (0.72-1.34)	.91	0.76 (0.19-3.00)	.70

The association between survival and T-AUC was corrected for potential confounders age, sex, history of coronary artery disease, witnessed arrest, time to basic life support (minutes), time to ROSC (minutes). Patients who had no information available on time to ROSC (per minute) could not be incorporated into the analysis (n = 67).

cTnT, troponin T; ROSC, return of spontaneous circulation ; T-AUC, time-concentration curves of cTnT.

a Effect sizes are depicted in hazard ratios.

b Effect sizes are depicted in odds ratios.

### Acute coronary lesions

The baseline cTnT, median cTnT, and T-AUC were all independently associated with acute unstable lesions (baseline cTnT unadjusted OR, 2.13; 95% CI, 0.92-4.91; *P* = .08; adjusted OR, 3.32; 95% CI, 1.11-9.94; *P* = .03; median cTnT unadjusted OR, 1.39; 95% CI, 1.11-1.73; *P* = .004; adjusted OR, 1.58; 95% CI, 1.18-2.12; *P* = .002; T-AUC unadjusted OR, 1.74; 95% CI, 1.20-2.53; *P* = .004; adjusted OR, 2.03; 95% CI, 1.25-3.29; *P* = .004), and acute thrombotic occlusions (median cTnT unadjusted OR, 1.55; 95% CI, 1.20-2.00; *P* < .001; adjusted OR, 1.62; 95% CI, 1.17-2.23; *P* = .003; T-AUC unadjusted OR, 2.18; 95% CI, 1.43-3.34; *P* < .001; adjusted OR, 2.16; 95% CI, 1.27-3.68; *P* = .004). Peak cTnT was not independently associated with acute thrombotic occlusions (unadjusted OR, 1.17; 95% CI, 1.02-1.34; *P* = .02; adjusted OR, 1.16; 95% CI, 0.99-1.36; *P* = .06), or with acute unstable lesions (unadjusted OR, 1.11; 95% CI, 0.99-1.24; *P* = .09; adjusted OR, 1.14; 95% CI, 0.99-1.30; *P* = .06. ROC curves for the performance of all cTnT values in the prediction of ACS are shown in Figure 2C and D.

### LVF

No correlation was found between LVEF and baseline cTnT, median cTnT, peak cTnT, and T-AUC (Spearman ρ = −0.03, −0.12, −0.07 and −0.012; *P* value = .74, .17, .42 and .88), respectively. Correspondingly, when LVEF was dichotomized to normal or abnormal, an abnormal LVF was not found to be associated with baseline cTnT (unadjusted OR, 1.54; 95% CI, 0.43-5.53; *P* = .51; adjusted OR, 1.89; 95% CI, 0.41-8.66; *P* = .41), the median cTnT (unadjusted OR, 2.06; 95% CI, 0.79-5.36; *P* = .14), peak cTnT (unadjusted OR, 1.11; 95% CI, 0.88-1.39; *P* = .38) or T-AUC (unadjusted OR, 2.01; 95% CI, 0.65-6.19; *P* = .22).

## Discussion

CTnT is currently routinely obtained in patients who are successfully resuscitated from cardiac arrest. In patients without ST-elevation after ROSC, a degree of uncertainty remains on the clinical value of these measures. The current study found that various measures of cTnT, obtained in the first 72 hours after ROSC, are associated with survival, neurologic outcome, and ACS among patients without ST-elevation. We studied the prognostic value of baseline cTnT, median cTnT, peak cTnT, and the time-concentration curve of cTnT. Using high-qualitative data from a large randomized trial, this study evaluated the prognostic value of serial cTnT values after cardiac arrest in patients without ST-segment elevation or other obvious extracoronary causes of the arrest.

### Clinical implication of cTnT measurements

The role of immediate coronary angiography in patients successfully resuscitated from OHCA, in the absence of STEMI has been debated for a long time. Recently, 4 randomized trials failed to show the benefit of a routine use of such an immediate invasive strategy.^4^^,^^5^^,^^7^^,^^15^ Current guidelines state that the management of postcardiac arrest non-STE-segment elevation ACS patients’ needs to be individualized.^16^ However, it does not guide how to translate this advice into clinical practice. Numerous studies have discussed how to identify patients with ACS who may benefit from an immediate invasive strategy.^17^, ^18^, ^19^, ^20^ To date, no isolated risk factors have been established yet. CTnT obtained directly after ROSC as a prognostic factor for survival^21^^,^^22^ or as a predictor for acute coronary lesions has performed poorly in previous studies.^23^^,^^24^ In this study, cTnT at baseline was independently associated with survival. Moreover, baseline cTnT had the largest hazard ratios for death compared to the other 3 types of measures. The AUC for baseline cTnT, however, was 0.57 and thus it seems that the first cTnT measurement cannot reliably predict a patient’s prognosis. Previous attempts to determine the cTnT threshold to predict acute coronary occlusions in postarrest patients have also failed, as studies repeatedly found that the positive predictive value of various cTnT cut-offs is low.^12^, ^13^, ^14^ Moreover, in patients who die shortly after ROSC, cTnT measurements obtained until that time may still be low, as cTnT generally peaks within hours to days after the arrest.^21^^,^^23^^,^^25^ Hence, as a major diagnostic criterion for type I myocardial infarction according to the fourth universal definition (ie, detection of a rise and/or fall of cTnT with at least 1 value above the 99th percentile of upper reference limit),^11^ its applicability in cardiac arrest patients has serious limitations.^20^ In addition to ACS, many other factors are associated with cTnT release during cardiac arrest. Some factors may be considered causative (eg, defibrillation) and some factors may increase the likelihood of cTnT increase (eg, age, sex, duration of resuscitation).^23^^,^^24^^,^^26^^,^^27^ Depending on the definition of the upper limit of normal cTnT, 92 to 100% of patients have elevated troponins after resuscitation.^13^^,^^14^^,^^25^^,^^28^^,^^29^ CTnT levels above the upper limit of normal should therefore, not be attributed solely to ACS, as it may also be a part of the global myocardial ischemia-reperfusion state.^28^^,^^30^

However, this study found that cTnT values were associated with acute unstable lesions and acute thrombotic occlusions, also after correction for potential confounders such as patient characteristics or arrest features. However, the performance of cTnT values in predicting acute unstable lesions was poor with AUC between 0.59 to 0.63. These results may suggest that cTnT values after OHCA are indeed predictive for ACS, but concurrently may not be distinctive enough for clinical utility.

Recently, Patel et al^31^ investigated the use of serial high-sensitivity cTnT in stabilized nonresuscitated ACS patients and found a 3-fold greater risk for cardiovascular events in patients with an increased cTnT in the first few months after admission. In this population of OHCA patients, however, its use for the prediction of events may have less clinical yield since the opportunity for immediate coronary angiography has passed when serial measurements are available. Nonetheless, these results might indicate that larger cTnT values may confirm the need for angiography at a later stage. Furthermore, serial cTnT measurements may still be of interest in support of the presumed cause of the arrest as they influence further therapy, such as the need for ICD implantation.

Since cTnT is a biomarker for myocardial tissue damage, one might expect an inverse association with LVF. This was found in a previous study that reported a significant association between the cTnT time-concentration curve and LVEF among nonresuscitated patients presenting with ACS.^32^^,^^33^ We did not find such results in this population of cardiac arrest patients without STEMI. To what extent the cause of the arrest may have influenced both LVF and cTnT release is unknown. Unfortunately, the preexistent LVF was not available, and therefore we do not know how this may have affected the results. However, a strategy of immediate or delayed coronary angiography did not affect LVF, as previously reported.^34^

An important challenge clinicians face in selecting patients for coronary angiography is identifying those with a high risk of ACS but a low risk of neurological death*.* Further research should be undertaken to investigate the added value of cTnT in the risk stratification of these patients. Potentially, combining patient characteristics and clinical findings, such as ECG findings and neurological prognosis, may enhance risk stratification and, in this way, accommodate the individual approach as implied in the guidelines^16^; this is however yet to be explored. Also, prospective studies showed promising results on the role of ECG-gated coronary computed tomography angiography in OHCA patients as a gatekeeper for coronary angiography and need further evaluation.^35^^,^^36^

### Limitations

We acknowledge the following limitations. Although obtaining cTnT in the first 72 hours of admission was part of the COACT trial protocol, in several patients, measurements were missing. Patients with 1 or no cTnT measurement available were not included in the analysis, which may have introduced potential bias. For patients who had missing values during the serial cTnT testing, the T-AUC was interpolated. Patients with cTnT measurements were not included in the analysis. And finally, the cTnT measurements were not performed in a central laboratory, but in the local laboratories of the participating centers.

## Conclusion

Although CTnT is currently routinely obtained in OHCA patients, there remains a degree of uncertainty in interpretation and its clinical implications. This study found that in OHCA patients without ST-segment elevation, cTnT release during the first 72 hours after ROSC was an independent predictor for survival, neurologic outcome, and acute unstable lesions. For the performance of cTnT in the prediction of ACS, higher values had a fair discrimination ability with AUC ranging from 0.72 to 0.81.
