# Supplementary material for: The Prognostic Value of Troponin-T in Out-of-Hospital Cardiac Arrest Without ST-Segment Elevation: A COACT Substudy
Source: J Soc Cardiovasc Angiogr Interv. 2023 Oct 14;3(2):101191. doi: 10.1016/j.jscai.2023.101191 (PMC11308418; doi:10.1016/j.jscai.2023.101191)
Supplement: Supplementary Data [file mmc1.docx]

**Supplementary appendix**

**The prognostic value of troponin T in out-of-hospital cardiac arrest patients without ST-segment elevation:**

**A secondary analysis of the COACT trial**

Eva M. Spoormans MD^1^, Jorrit S. Lemkes MD, PhD^1^, Gladys N. Janssens MD, PhD^1^, Nina W. van der Hoeven MD, PhD^1^, Lucia S.D. Jewbali MD^2^, Eric A. Dubois MD, PhD^2,3^, Martijn Meuwissen, MD, PhD^4^, Tom A. Rijpstra, MD, PhD^5^, Hans A. Bosker, MD, PhD^6^, Michiel J. Blans, MD, PhD^7^, Gabe B. Bleeker, MD, PhD^8^, Remon Baak, MD^9^, Georgios J. Vlachojannis, MD, PhD^10,11^, Bob J.W. Eikemans, MD^12^, Pim van der Harst, MD, PhD^11,13^, Iwan C.C. van der Horst, MD, PhD^14,15^, Michiel Voskuil,

MD, PhD^11^, Joris J. van der Heijden, MD^16^, Albertus Beishuizen, MD, PhD^17^, Martin Stoel, MD, PhD^18^, Cyril Camaro, MD, PhD^19^, Hans van der Hoeven, MD, PhD^20^, José P. Henriques, MD, PhD^21^, Alexander P.J. Vlaar, MD, PhD^22^, Maarten A. Vink, MD, PhD^23^, Bas van den Bogaard, MD, PhD^24^, Ton A.C.M. Heestermans, MD, PhD^25^, Wouter de Ruijter, MD, PhD^26^, Thijs S.R. Delnoij, MD, PhD^27^, Harry J.G.M. Crijns, MD, PhD^28^, Pranobe V. Oemrawsingh, MD, PhD^29^, Marcel T.M. Gosselink, MD, PhD^30^, Koos Plomp MD^31^, Michael Magro, MD, PhD^32^, Paul W.G. Elbers, MD, PhD^33^, Stéphanie van der Pas, PhD^34,35^, Niels van Royen, MD, PhD^1, 19^

1 Department of Cardiology, Amsterdam University Medical Center, location VUmc, Amsterdam, the Netherlands.

2 Department of Cardiology, Erasmus Medical Center, Rotterdam, the Netherlands.

3 Department of Intensive care medicine, Erasmus Medical Center, Rotterdam, the Netherlands

4 Department of Cardiology, Amphia Hospital, Breda, the Netherlands.

5 Department of Intensive care medicine, Amphia Hospital, Breda, the Netherlands.

6 Department of Cardiology, Rijnstate Hospital, Arnhem, the Netherlands.

7 Department of Intensive care medicine, Rijnstate Hospital, Arnhem, the Netherlands.

8 Department of Cardiology, HAGA Hospital, Den Haag, the Netherlands.

9 Department of Intensive care medicine, HAGA Hospital, Den Haag, the Netherlands.

10 Department of Cardiology, Maasstad Hospital, Rotterdam, the Netherlands.

11 Department of Cardiology, University Medical Center Utrecht, Utrecht, the Netherlands

12 Department of Intensive care medicine, Maasstad Hospital, Rotterdam, the Netherlands.

13 Department of Cardiology, University Medical Center Groningen, Groningen, the Netherlands.

14 Department of Intensive care medicine, University Medical Center Groningen, Groningen, the Netherlands.

15 Department of Intensive care medicine, Maastricht University Medical Center, Maastricht, the Netherlands.

16 Department of Intensive care medicine, University Medical Center Utrecht, Utrecht, the Netherlands.

17 Intensive Care Center, Medisch Spectrum Twente, Enschede, The Netherlands.

18 Department of Cardiology, Medisch Spectrum Twente, Enschede, The Netherlands.

19 Department of Cardiology, Radboud University Medical Center, Nijmegen, the Netherlands.

20 Department of Intensive care medicine, Radboud University Medical Center, Nijmegen, the Netherlands.

21 Department of Cardiology, Amsterdam University Medical Center, location AMC, Amsterdam, the Netherlands.

22 Department of Intensive care medicine, Amsterdam University Medical Center, location AMC, Amsterdam, the Netherlands.

23 Department of Cardiology, OLVG, Amsterdam, the Netherlands.

24 Department of Intensive care medicine, OLVG, Amsterdam, the Netherlands.

25 Department of Cardiology, Noord West Ziekenhuisgroep, Alkmaar, the Netherlands.

26 Department of Intensive care medicine, Noord West Ziekenhuisgroep, Alkmaar, the Netherlands.

27 Department of Intensive care medicine, Maastricht University Medical Center, Maastricht, the Netherlands.

28 Department of Cardiology, Maastricht University Medical Center, Maastricht, the Netherlands.

29 Department of Cardiology, Haaglanden Medical Center, Den Haag, the Netherlands.

30 Department of Cardiology, Isala Hospital, Zwolle, the Netherlands.

31 Department of Cardiology, Tergooi Hospital, Blaricum, the Netherlands.

32 Department of Cardiology, Elisabeth-Tweesteden Hospital, Tilburg, the Netherlands.

33 Department of Intensive care medicine, Amsterdam University Medical Center, location VUmc, Amsterdam, the Netherlands.

34 Epidemiology and Data Science, Amsterdam University Medical Center, location Vrije Universiteit Amsterdam, Amsterdam, Netherlands

35 Amsterdam Public Health, Methodology, Amsterdam, The Netherlands

**Index**

Definitions 3

Inclusion and exclusion criteria of the COACT trial 4

Tables

Table S1: The association between cTnT and primary and secondary 5

outcomes in all patients (including outliers)

Figures

Figure S1: Study flowchart 6

Figure S2: Frequency distribution of troponin-T AUC 7

References 8

**Definitions**

Acute unstable lesions

All coronary lesions with a stenosis severity of ≥70% and the presence of characteristics of plaque disruption including lesion irregularity, dissection, haziness or thrombus defined by coronary angiography.

Neurologic outcome according to the Cerebral Performance Category (CPC) scale

CPC 1: Full recovery or mild disability.

CPC 2: Moderate disability but independent in activities of daily living.

CPC 3: Severe disability: dependent in activities of daily living.

CPC 4: Persistent vegetative state.

CPC 5: Dead.

**Inclusion and exclusion criteria of the COACT trial** (1)

Inclusion criteria

- Age > 18 years
- Comatose patients (Glasgow coma score < 8) with return of spontaneous circulation after out-of-hospital cardiac arrest.
- Ventricular tachycardia or ventricular fibrillation as initial arrest rhythm. Including patients treated with an AED.

Exclusion criteria

- Signs of STEMI on the ECG at the emergency department (including new LBBB or isolated ST depression in V1-V3 due to a true posterior infarct).
- Hemodynamic instability unresponsive to medical therapy. Defined as a prolonged (>30 min) systolic blood pressure <90 mmHg at the time of screening.
- An obvious or suspected non-coronary cause of the arrest.
- A known severe renal dysfunction (GFR<30 ml/min).
- Obvious or suspected pregnancy.
- Suspected or confirmed acute intracranial bleeding.
- Suspected or confirmed acute stroke.
- Known limitations in therapy or DO Not Resuscitate-order.
- Known pre-arrest Cerebral Performance Category 3 or 4.
- >4 hours (from return of spontaneous circulation to screening).
- Refractory ventricular arrhythmia.
- Known inability to complete 90-day follow-up

|  | **Baseline cTnT** | | | **Median cTnT** | | | **Peak cTnT** | | | **T-AUC** | | |
| --- | --- | --- | --- | --- | --- | --- | --- | --- | --- | --- | --- | --- |
|  | **Effect size (95% CI)** | **p-value** | **AUROC** | **Effect size (95% CI)** | **p-value** | **AUROC** | **Effect size (95% CI)** | **p-value** | **AUROC** | **Effect size (95% CI)** | **p-value** | **AUROC** |
| Survival | 1.61 (1.13 – 2.29) | 0.009 | 0.57 | 0.99 (0.97 – 1.02) | 0.58 | 0.63 | 0.99 (0.98 – 1.01) | 0.40 | 0.62 | 0.96 (0.88 – 1.05) | 0.41 | 0.61 |
|  | 2.01 (1.43 – 2.82) | <0.001 |  | 1.00 (0.97 – 1.03) | 0.98 |  | 0.99 (0.98 – 1.01) | 0.43 |  | 0.96 (0.85 – 1.08) | 0.46 |  |
| Poor neurological outcome | 1.39 (0.81 – 2.39) | 0.24 | 0.57 | 0.99 (0.97 – 1.02) | 0.54 | 0.63 | 0.99 (0.98 – 1.01) | 0.36 | 0.61 | 0.96 (0.88 – 1.05) | 0.38 | 0.62 |
|  | 2.21 (0.85 – 5.72) | 0.10 |  | 0.99 (0.96 – 1.04) | 0.91 |  | 0.99 (0.97 – 1.01) | 0.40 |  | 0.95 (0.84 – 1.08) | 0.42 |  |
| Acute unstable lesions | 1.97 (0.94 – 4.15) | 0.07 | 0.63 | 1.01 (1.00 – 1.02) | 0.15 | 0.63 | 1.00 (1.00 – 1.01) | 0.81 | 0.59 | 0.99 (0.96 – 1.03) | 0.76 | 0.61 |
|  | 2.64 (1.01 – 6.94) | 0.049 |  | 1.00 (0.96 – 1.04) | 0.94 |  | 0.99 (0.97 – 1.02) | 0.55 |  | 0.98 (0.89 – 1.07) | 0.64 |  |
| Acute thrombotic occlusions | 2.03 (1.01 – 4.05) | 0.046 | 0.73 | 1.01 (1.00 – 1.02) | 0.06 | 0.79 | 1.00 (1.00 – 1.00) | 0.18 | 0.77 | 1.01 (0.98 – 1.04) | 0.74 | 0.81 |
|  | 2.33 (0.99 – 5.45) | 0.052 |  | 1.01 (0.96 – 1.06) | 0.78 |  | 1.00 (0.97 – 1.02) | 0.72 |  | 0.99 (0.91 – 1.09) | 0.88 |  |
| Abnormal left ventricular function | 1.76 (0.50 – 6.19) | 0.38 | 0.50 | 0.98 (0.93 – 1.02) | 0.30 | 0.56 | 1.00 (1.00 – 1.00) | 0.94 | 0.56 | 1.00 (0.98 – 1.02) | 0.89 | 0.52 |
|  | 2.06 (0.47 – 9.13) | 0.34 |  | 0.95 (0.86 – 1.05) | 0.29 |  | 1.00 (0.996 – 1.01) | 0.73 |  | 1.00 (0.97 – 1.02) | 0.75 |  |

**Table S1: The association between cTnT and primary and secondary outcomes in all patients (including outliers)**

Baseline cTnT, median cTnT, peak cTnT and T-AUC and its association with each outcome. Per outcome, the upper row represents the unadjusted effect size (95% CI) and second row represents the adjusted effect size (95% CI). The association between survival and T-AUC was corrected for potential confounders age, sex, history of coronary artery disease, witnessed arrest, time to basic life support (min), time to return of spontaneous circulation (min). Patients that had no information available on time to ROSC (per minute) could not be incorporated in the analysis (n=67). Abbreviations: CI confidence interval.

**
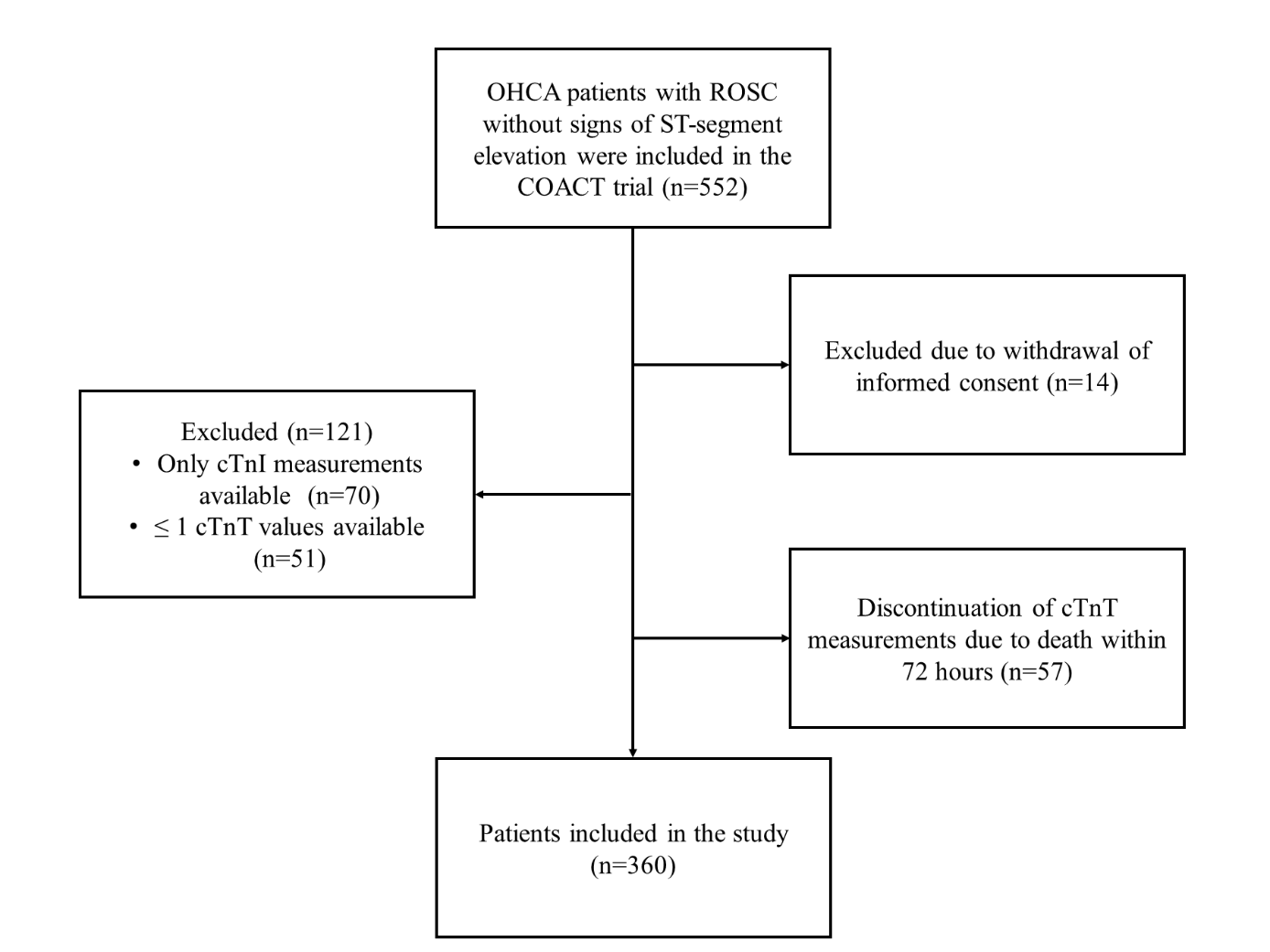
**

**Figure S1**: Study flowchart

**
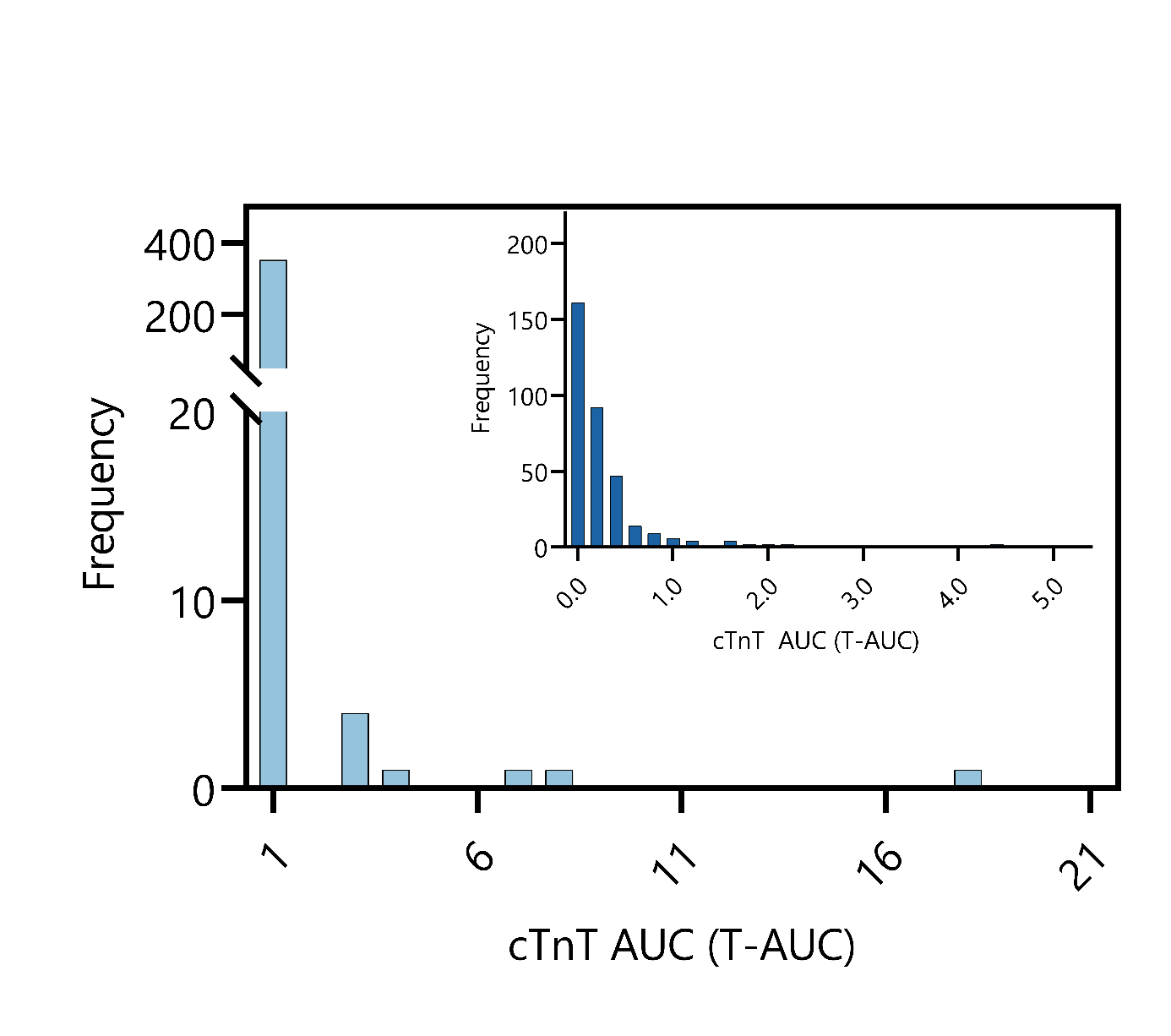

Figure S2**: Frequency distribution of cTnT Area Under the Curve (T-AUC) of the 360 patients in whom a T-AUC could be calculated.

**References**
1. Lemkes JS, Janssens GN, van der Hoeven NW, Jewbali LSD, Dubois EA, Meuwissen M, et al. Coronary Angiography after Cardiac Arrest without ST-Segment Elevation. N Engl J Med. 2019;380(15):1397-407.
